# MiR-9-5p promotes rabbit preadipocyte differentiation by suppressing *leptin* gene expression

**DOI:** 10.1186/s12944-020-01294-8

**Published:** 2020-06-05

**Authors:** Gang Luo, Shenqiang Hu, Tianfu Lai, Jie Wang, Li Wang, Songjia Lai

**Affiliations:** Farm Animal Genetic Resources Exploration and Innovation Key Laboratory of Sichuan Province, Sichuan Agricultural University, 211#Huimin Road, Wenjiang, Chengdu, 611130 Sichuan China

**Keywords:** MiR-9-5p, Rabbits, Pre-adipocyte, *Leptin*, Differentiation

## Abstract

**Background:**

MicroRNAs (miRNAs) are a class of small non-coding RNAs, which participate in the regulation of cell differentiation. Previous studies have demonstrated that miR-9-5p plays a key role in cancer cell development, but the mechanisms by which miR-9-5p regulates adipogenesis remain poorly understood. The present study intended to investigate its significance in producing rabbits with high-quality meat by observing the regulatory effect of miR-9-5p in preadipocytes and finding the related targets.

**Methods:**

In this study, a dual-luciferase reporter assay was employed to validate the targeting relationship between miR-9-5p and *leptin* gene. We also utilized quantitative reverse transcription PCR (qRT-PCR), western blot, oil red-O staining assay, and determination of triglyceride content to analyze the regulation of miR-9-5p and *leptin* gene during adipocyte differentiation.

**Results:**

The analysis demonstrated that during preadipocyte differentiation, miR-9-5p was up-regulated and the fat formation related biomarkers, i.e., fatty acid-binding protein 4 (*FABP4*), *CCAAT*-enhancer binding protein α (*C/EBPα*), and peroxisome proliferator activated receptor γ (*PPARγ*) were also up-regulated. Meanwhile, the oil red-O staining assay revealed that the accumulation of lipid droplets increased. We also explored the expression pattern and role of miR-9-5p in adipogenesis using white pre-adipocytes. The results showed that miR-9-5p was up-regulated during preadipocyte differentiation, and overexpression of miR-9-5p enhanced lipid accumulation. Furthermore, we found that the overexpression of miR-9-5p significantly up- regulated the expression of marker genes, *PPARγ*, *C/EBPα* and *FABP4*, and increased the protein levels of *PPARγ* and triglyceride content. The results suggest that miR-9-5p might be involved in the regulation of rabbit preadipocyte differentiation. We predicted that *leptin* is the target gene of miR-9-5p, by using bioinformatics tools and the conclusion was validated by a luciferase reporter assay. Finally, we verified that the knock-down of *leptin* by si-*leptin* promoted preadipocyte differentiation in rabbits.

**Conclusion:**

The results of the present study indicate that miR-9-5p regulates white preadipocyte differentiation in rabbits by targeting the *leptin* gene.

## Introduction

Adipose tissue, which may be brown, beige, or white, not only plays an important role in energy metabolism by generating heat and storing lipids but also serve as an endocrine organ. White adipose tissue is widely dispersed and has the ability to differentiate. Adipose tissue-derived stem cells (ASCs) are stimulated to undergo transformation into preadipocytes, which in turn rapidly mature to adipocytes [10]. Adipogenesis, is a complex process in which fibroblast-like preadipocytes differentiate into lipid-laden and insulin-responsive adipocytes [18]. This process requires sequential activation of numerous transcription factors and noncoding RNAs. Several transcription factors such as peroxisome proliferator-activated receptor-γ (*PPARγ*) and members of the CCAAT/enhancer-binding family of proteins (*C/EBPα*) play a significant role in the regulation of adipogenesis [12, 28]. Expression of *FABP4* is induced during adipocyte differentiation and is controlled by insulin and *PPARγ* agonists [15]. *Leptin* plays a pivotal role in regulating food intake, energy expenditure, and fat deposition in mammals [20]. It can promote lipolysis and thermogenesis by influencing the sympathetic nervous system [6]. In addition, it can also directly regulate the metabolism of some tissues and cells [21, 32]. In a study conducted by Thierry et al. [36], *leptin* resulted in a dose and time-dependent increase in type1 collagen, a decrease of adipsin and *leptin* mRNA levels, and a 50% decrease in lipid droplet formation. Zhou et al. [43] reported fat loss, downregulation of lipogenic enzymes, and suppression of the transcription factor *PPARγ* caused by adenovirus-induced hyperleptinemia in normal rats. The absence of fat in transgenic mice led to a reduction in *leptin* levels and marked metabolic alterations including hyperlipidemia, insulin resistance, and diabetes [22]. Recent literature shows that *leptin* enhances the differentiation of osteoblasts and inhibits the differentiation of adipocytes in human bone marrow stromal cells [36]. Altogether, these findings indicate that *leptin* may act locally in adipose tissue to influence the formation of mature adipocytes.

Furthermore, microRNAs (miRNAs), a class of short noncoding RNAs, are involved in the inhibition of gene expression by binding to the 3′-UTRs of the target mRNAs and have been demonstrated to regulate lipid metabolism [4, 17]. miRNAs have also been shown to be involved in cell differentiation [41]. In goats, miR-183 promotes preadipocyte differentiation by suppressing the expression of Smad4 [42] and miR-21a-5p induces preadipocyte differentiation by targeting Mitogen-activated protein kinase 2/kinase 3 (*MAPK2K3)* through *MKK3/p38/MAPK* signaling pathway in mice [38]. MiR-33b downregulates the differentiation of porcine preadipocytes [35]. MiR-26b promotes adipocyte differentiation in 3 T3-L1 cells, through targeting *PTEN* [19], another study has demonstrated that miR-148a-3p promotes rabbit adipocyte differentiation by targeting *PTEN* in rabbits preadipocytes [14]. MiR-9-5p is a highly conserved miRNA that is primarily expressed in the central nervous system [16, 37]. A study conducted by Li et al. [19] showed that miR-9-5p can promote mesenchymal stem cell (MSC) migration by activating β-catenin signaling pathway. β-catenin plays an important role in the transduction of Wnt signals. Ross et al. [31] found that the classical Wnt signal can inhibit fat differentiation. It has also been shown that *C/EBPα* or *PPARγ* can inhibit Wnt signaling, which in turn can affect the differentiation of adipocytes by reducing the level of β-catenin [23]. Yet, the role of miR-9-5p in differentiation of adipocytes remains unclear.

In the present study, we isolated and cultured rabbit perirenal adipose cells. Then, we studied the effect of overexpression or inhibition of miR-9-5p on adipocyte differentiation. In addition, we also predicted and verified that *leptin* is the target gene of miR-9-5p. Finally, we found that miR-9-5p can influence the differentiation of adipocytes by downregulating the expression of *leptin* gene.

## Material and methods

### Animal and tissue collection

All the experimental procedures using rabbits in this study were conducted under a protocol approved by the Institutional Animal Care and Use Committee, in the College of Animal Science and Technology, Sichuan Agricultural University, China. The rabbits were killed by neck folding. Perirenal adipose tissues were collected from 3 newborn New Zealand rabbits, which were raised under standard conditions at the Sichuan Agricultural University farm (Yaan, Sichuan, China).

### Cell isolation, culture, and induction of adipogenesis

Adipose tissue was rinsed thrice with PBS and then minced and digested with 0.25% collagenase type I (Gibco, Carlsbad, CA, USA) at 37 °C for 1 h. The mixture was then added to the complete medium (CM; DM/F12, 10% fetal bovine serum, 2% penicillin-streptomycin) (Gibco) followed by filtration through 70-nm and 40-nm cell sieves, respectively. The final mixture was centrifuged at 1200×g for 5 min to collect the preadipocytes. The preadipocytes were then seeded into a culture flask with complete medium and incubated at 37 °C in a humidified incubator with 5% CO_2_. Culture medium was changed every 2 days and the cells were frozen for future studies. After the cells reached about 70% confluence, an adipogenic cocktail (0.5 mM 3-isobutyl-1-methylxanthine, 10% FBS, 1 μM dexamethasone, and 1.7 μM insulin) was added into the growth medium to induce differentiation. The cells were further incubated for 72 h, after which the medium was replaced with maintenance medium (growth medium supplemented with 1.7 mM insulin per 50 mL) and incubated for an additional 72 h. Thereafter, the cells were cultured in a growth medium until maturation at 10 days.

### Cell transfection

Preadipocyte cells were seeded into 24-well or 6-well plates and transfected using Lipofectamine 3000 (Invitrogen, Carlsbad, CA, USA) according to the manufacturer’s instructions after the cells had reached 70% confluency. The final concentrations of the negative control miRNA mimics (NC miR-Mimic), negative control inhibitor (inhibitor NC), and negative control *leptin* siRNA (siRNA NC) were 50 nM, 100 nM, and 100 nM, respectively. Three independent repetitions were performed for each treatment group. The cells were harvested at different time intervals after transfection and used to study adipogenic differentiation.

### RNA isolation and qPCR analysis

Total cellular RNA was extracted using RNAiso Plus reagent (TaKaRa, Shiga, Japan) following manufacturer’s instructions. The concentration and quality of the RNA was evaluated using Nano Drop 2000 UV-Vis Spectrophotometer (Thermo Scientific, Waltham, MA, USA). RNA purity was evaluated by determining the ratios of absorbance at 260 nm and 280 nm (A260/A280) and at 260 nm and 230 nm (A260/A230). First-strand cDNA of total RNA and small RNA was synthesized using the Prime Script RT reagent Kit (Takara, Japan) and the SYBR® Prime Script™ miRNA reverse transcription Kit (Takara), respectively, according to the manufacturer’s protocol. The corresponding cDNA was stored at − 20 °C. qPCR was performed using SYBR premix Ex Taq II (Tli RNase H Plus) (Catalog No. RR820A; Takara) on CFX96 system (Bio-Rad, Hercules, CA, USA). U6 was selected to normalize the expression of miRNA, while the internal reference used for mRNA expression was actin. The RT-qPCR procedure was as follows: Initial denaturation for 3 min at 95 °C, 39 cycles of denaturation at 95 °C for 30 s, annealing at the optimum temperature for 30 s, and final extension for 66 s with temperature increments of 0.5 °C/s from 65 °C to 95 °C. The 2^−ΔΔCt^ method was used to analyze the relative expression of each gene. The sequences of the primers used for qPCR are shown in Table 1.

**Table 1:** Primers used in this study

Gene name	Primer sequence (5′-3′)	(Tm/°C)	(Product size/bp)
*leptin*	CTGTGCCCATGCGGAAAGTC	61.4	104
	AGTCCAAACCGACGACCCTC		
*β-actin*	GGAGATCGTGCGGGACAT	61.4	318
	GTTGAAGGTGGTCTCGTGGAT		
*PPARγ*	GAGGACATCCAGGACAACC	61	168
	GTCCGTCTCCGTCTTCTTT		
*FABP4*	GGCCAGGAATTTGATGAAGTC	61.4	140
	AGTTTATCGCCCTCCCGTT		
*C/EBPα*	GCGGGAACGAACAACAT	64	172
	GGCGGTCATTGTCACTGGTC		
miR-9-5p mimic	UCUUUGGUUAUCUAGCUGUAUGA AUACAGCUAGAUAACCAAAGAUU		
Negative Control (NC)	UUCUCCGAACGUGUCACGUTT ACGUGACACGUUCGGAGAATT		
inhibitor-9-5p	UCAUACAGCUAGAUAACCAAAGA		
inhibitor NC	CAGUACUUUUGUGUAGUACAA		
si-*leptin*-1	CCACAAUGGACCAGACGUUTT AACGUCUGGUCCAUUGUGGTT		
si-*leptin*-2	CCAUUGUCACCAGGAUCAGTT CUGAUCCUGGUGACAAUGGTT		
si-*leptin*-3	CCGAAAUGUGAUCCAAAUATT UAUUUGGAUCACAUUUCGGTT		
Primers for gene 3’UTR	ATGGGTAAGTACATCAAGAG		
cloning *leptin*	GAGGTCCGAAGACTCATTT		

### Western blotting

Total protein from the cell samples was extracted with the Total Protein Extraction Kit (Sangon, Shanghai, China) following the manufacturer’s protocol and protein content was quantified with the BCA Protein Assay Kit. Proteins (40 mg) were resolved on 8–12% SDS-polyacrylamide gels and transferred to a PVDF membrane (Bio-Rad). The PVDF membrane was rinsed with TBS-Tween20 (TBST) and blocked for 2 h in skimmed milk. Then, the membranes were incubated overnight with anti-*PPARγ* (Santa Cruz Biotechnology,) and anti-β-actin (Abs, Beijing, China) according to the respective instructions. The PVDF membranes were then washed thrice and incubated with the secondary goat anti-mouse IgG (H + L) (Transgen, Illkirch-Graffenstaden, France) for 2 h. The protein bands were incubated with chemiluminescence reagents (Beyotime) after being washed four times. The images were obtained with a Bio-Rad GelDoc system equipped with a Universal Hood III (Bio-Rad), and the integrated optical density (IOD) was calculated using Gel-Pro Analyzer 4.0.0.4. Actin was used as an internal control.

### Oil red-O staining and determination of triglyceride content

Adipocytes were washed thrice with phosphate-buffered saline (PBS) and fixed in 4% paraformaldehyde for 30 min. The fixed cells were stained with 1% filtered oil red-O solution for about 30 min in the dark. The adipocytes were then washed and observed under a phase contrast microscope. Finally, oil red-O was eluted from the stained cells with isopropanol and quantified by measuring the optical density values at 510 nm wavelength. Intracellular triglyceride (TG) content was quantified with the TG Assay Kit (Applygen, Beijing, China) according to the manufacturer’s protocol. The TG content (nmol/mg protein) was normalized to the cellular protein concentration, and the protein concentrations were measured with the BCA Protein Assay Kit (Beyotime, Shanghai, China).

### Prediction and verification of the target gene of miR-9-5p

MiRwalk (http://zmf.umm.uni-heidelberg.de/apps/zmf/mirwalk2/) was employed to predict the target genes of miR-9-5p. *Leptin* was found to be the target gene of miR-9-5p. The 3’UTR of *leptin* containing the miR-9-5p target site was cloned into the Sac I-Xba I site of the pmirGLO Vector (Promega, Madison, WI) to construct luciferase reporter plasmids. The primers used for plasmid construction are listed in Table 1. Multiple cloning sites were located downstream of firefly luciferase gene. HeLa cells were seeded into 24-well plates in triplicate. Then, the pmirGLO-*leptin*-3′ UTR of wild or mutant type was co-transfected with the synthetic miR-9-5p mimic into HeLa cells after the cell density reached 70–80%. Firefly luciferase (luc2) activity was measured 48 h after transfection and normalized to Renilla luciferase activity according to the TransDetect® Double Luciferase Reporter Assay Kit instructions (Transgen, Beijing, China).

### Statistical analysis

The RT-qPCR results of mRNA expression were normalized to the geometric average of *Actin* identified stability, and the expression level of miRNA was normalized to the expression of U6 snRNA, by an optimized comparative Ct (2 − ΔΔCt) value method. The entire data is presented as mean ± standard error (SEM). The student’s t-test in GraphPad Prism7 (GraphPad Software, La Jolla, CA, USA) was used to assess the difference between groups. *P* < 0.05 and *P* < 0.01 were deemed to be significant and highly significant, respectively.

## Results

### Expression profiling of miR-9-5p during rabbit preadipocyte differentiation

**Fig. 1:**
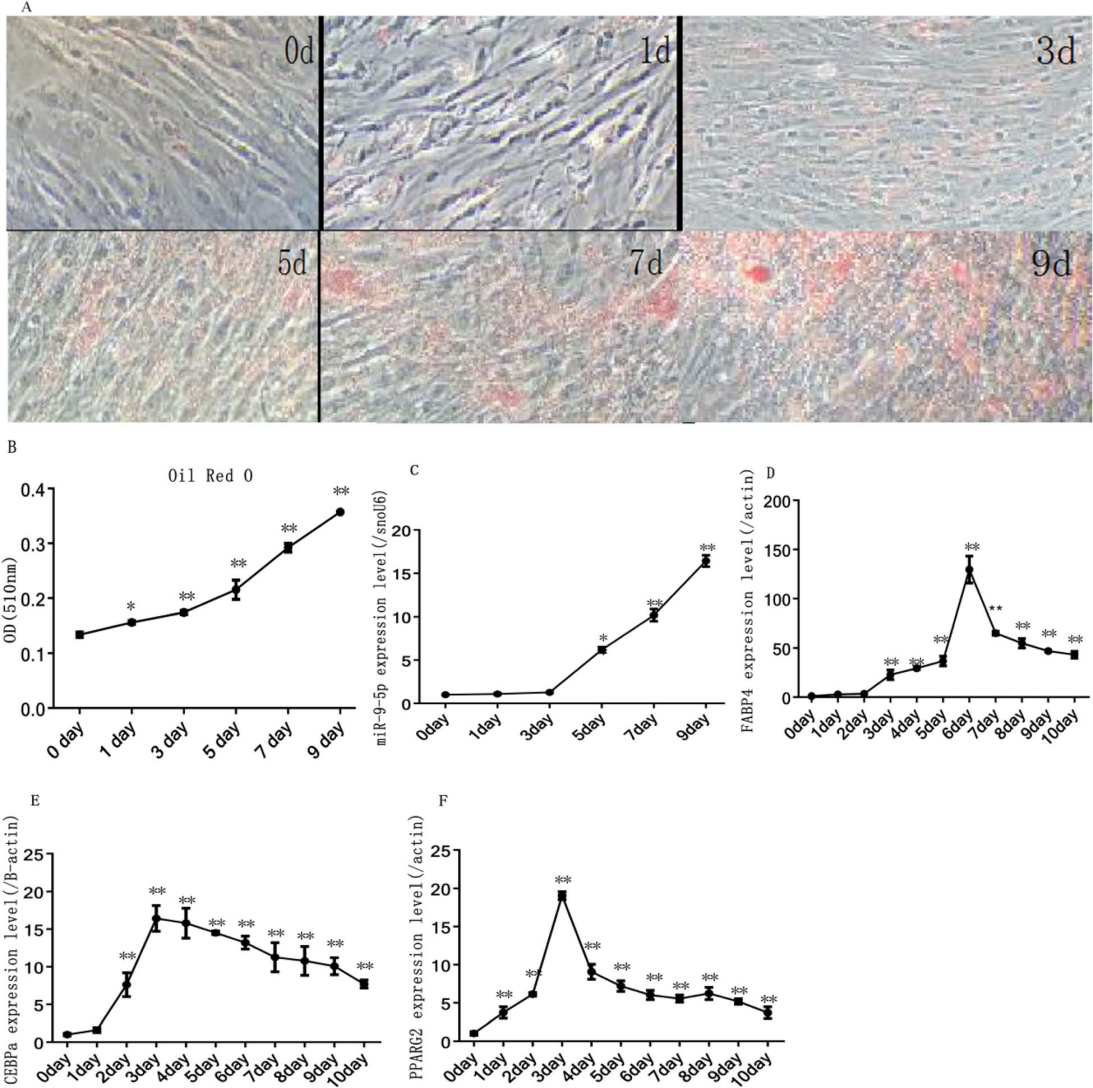
Rabbit preadipocyte differentiation model (**a**) Oil Red-O staining of lipid droplets; (**b**) quantitative detection of oil red O staining; (**c**) MiR-9-5p expression levels during preadipocyte differentiation under normal culture conditions; (**d**) *FABP4* expression levels during preadipocyte differentiation under normal culture conditions; (**e**) *CEBPα* mRNA expression levels during preadipocyte differentiation under normal culture conditions; (**f**) *PPARγ* expression levels during preadipocyte differentiation under normal culture conditions (“*”, *P* ≤ 0.05; “**”, *P* ≤ 0.01)

In the process of inducing differentiation, the adipocytes reached full differentiation, depositing large lipid droplets (Fig. 1a). Results of oil red-O staining showed that lipid droplets rapidly increased during preadipocyte cell differentiation (Fig. 1b). Simultaneously, the expression of miR-9-5p increased rapidly after 3 days of induction and peaked on the ninth day (Fig. 1c). Correspondingly, adipogenic marker gene *FABP4* had the highest mRNA expression on the sixth day whereas *PPARγ* and *CEBPα* had the highest expression on the third day after induction (Fig. 1d, e, f).

### Upregulation of miR-9-5p promotes rabbit preadipocyte differentiation

**Fig. 2:**
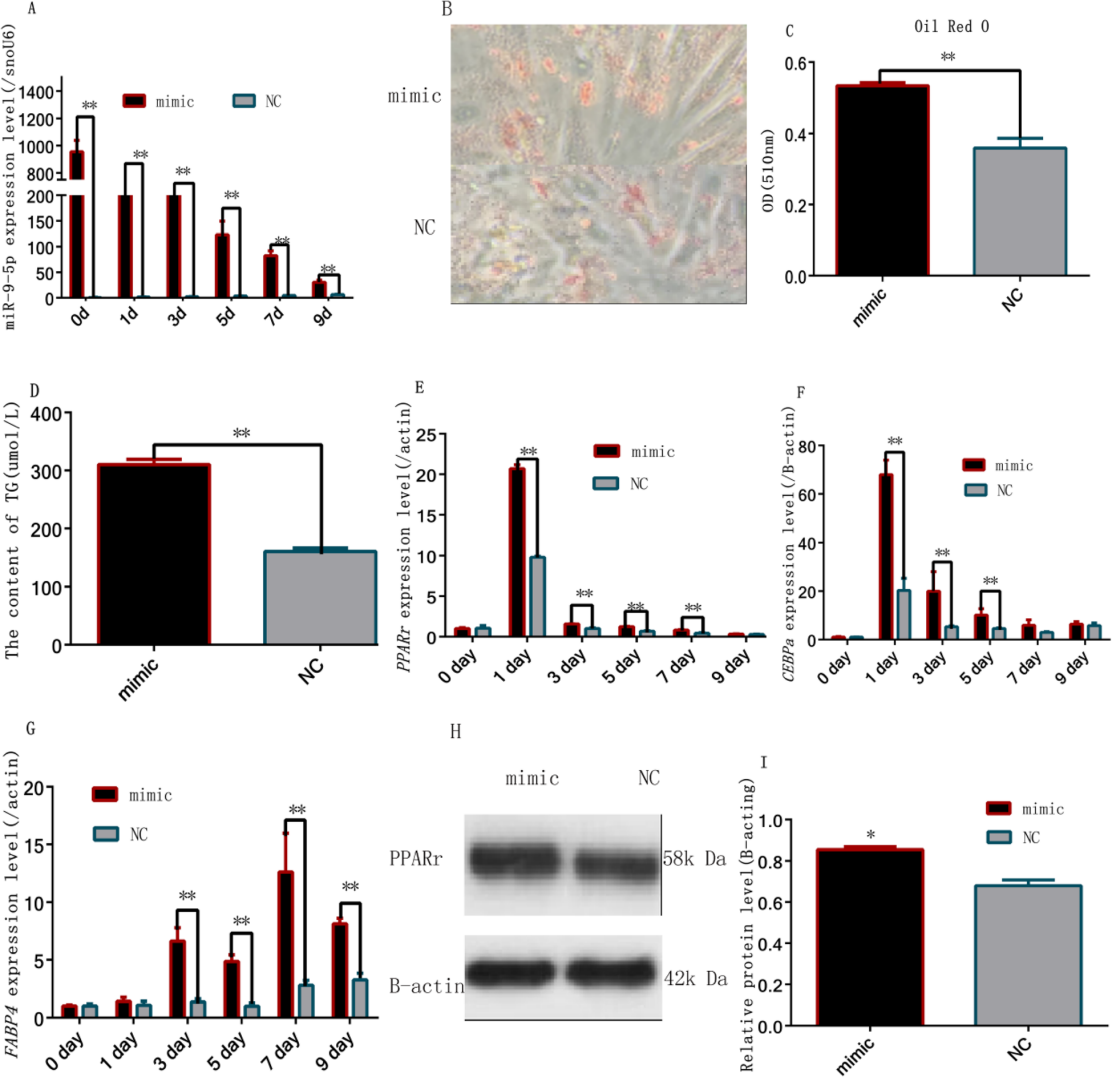
Overexpression of miR-9-5p promoted rabbit preadipocyte differentiation (**a**) MiR-9-5p expression levels during preadipocyte differentiation after transfecting with miR-9-5p mimics and NC, (**b**) Oil Red-O staining of lipid droplets on day 9; (**c**) Quantitative detection of oil red O staining on day 9; (**d**) Accumulation of triacylglycerol on day 9; (**e**) *PPARγ* expression levels during preadipocyte differentiation after transfecting with miR-9-5p mimics and NC; (**f**) *CEBPα* expression levels during preadipocyte differentiation after transfecting with miR-9-5p mimics and NC; (**g**) *FABP4* expression levels during preadipocyte differentiation after transfecting with miR-9-5p mimics and NC (**h**, **i**) *PPARγ* protein levels during preadipocyte differentiation after transfecting with miR-9-5p mimics and NC (“*”, *P* ≤ 0.05; “**”, *P* ≤ 0.01)

In order to investigate the function of miR-9-5p in rabbit preadipocyte cell differentiation, we first detected the expression level of miR-9-5p in the cells transfected with miR-9-5p mimics and NC during differentiation. After induction, miR-9-5p showed a higher expression level in the mimic group than in the NC group (*P* < 0.01) (Fig. 2a). Oil red-O staining results (including quantitative detection; *P* < 0.01) showed that the lipid accumulation was higher in the mimic group as compared with the NC group (Fig. 4b, c). In addition, there was a significant increase in triglycerides owing to the overexpression of miR-9-5p (Fig. 2d). The mRNA levels of adipogenic markers including *PPARγ* and C/EBPα were significantly higher in the mimic group than NC group on the first and third days (*P* < 0.01) (Fig. 2e, f, and g). Western blot analysis further indicated that *PPARγ* protein levels were significantly higher in the mimic group than NC group after 2 days of transfection (*P* < 0.05) (Fig. 2h, i).

### Downregulation of miR-9-5p inhibited rabbit preadipocyte differentiation

**Fig. 3:**
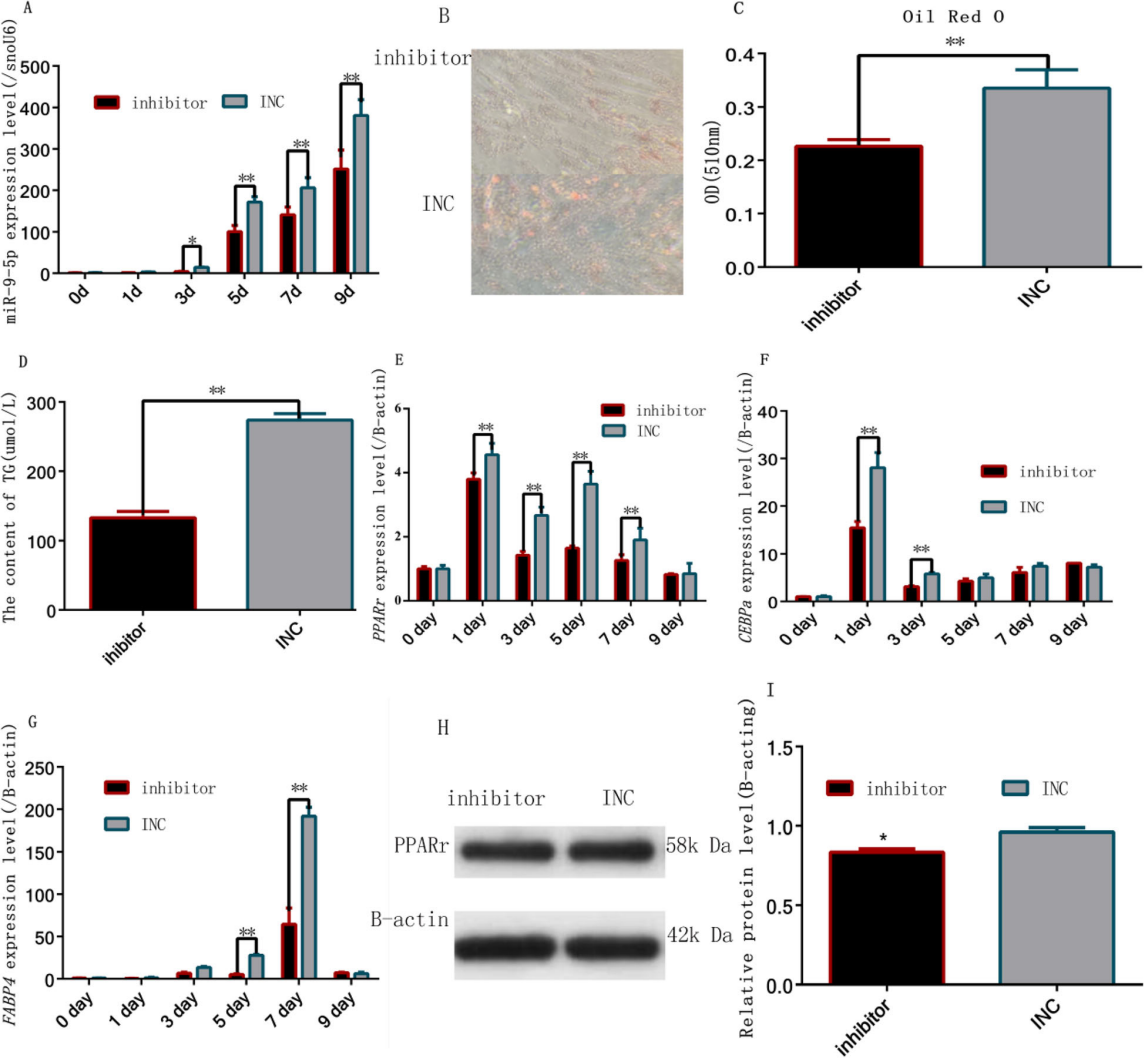
Inhibition of miR-9-5p inhibited rabbit preadipocyte differentiation (**a**) MiR-9-5p expression levels during preadipocyte differentiation after transfecting with the miR-9-5p inhibitor and INC, (**b**) Oil Red-O staining of lipid droplets on day 9; (**c**) Quantitative detection of oil red O staining on day 9; (**d**) Accumulation of triacylglycerol on day 9; (**e**) *PPARγ* expression levels during preadipocyte differentiation after transfecting with the miR-9-5p inhibitor and INC; (**f**) *CEBPα* expression levels during preadipocyte differentiation after transfecting with the miR-9-5p inhibitor and INC; (**g**) *FABP4* expression levels during preadipocyte differentiation after transfecting with the miR-9-5p inhibitor and INC (**h**, **i**) *PPARγ* protein levels during preadipocyte differentiation after transfecting with the miR-9-5p inhibitor and INC (“*”, *P* ≤ 0.05; “**”, *P* ≤ 0.01)

Next, we examined the differentiation of preadipocytes when endogenous miR-9-5p was effectively inhibited with the miR-9-5p inhibitor. As shown in Fig. 3a, the expression levels in the inhibitor groups were significantly lower than in the NC group on third, 5th, 7th, and 9th day after transfection (*P* < 0.01). We also found that down-regulation of miR-9-5p decreases lipid accumulation in the preadipocytes cells as detected by oil red-O staining (Fig. 3b, c) and TG content (Fig. 3d). The mRNA expression of the common differentiation-related genes (*PPARγ* and *C/EBPα*) was lower in the inhibition groups on the first and third day after transfection (Fig. 3e, f), whereas the expression of *FABP4* (another common differentiation-related gene) was lower on the fifth and seventh day after transfection in the inhibition groups (*P* < 0.01) (Fig. 3g). In addition, *PPARγ* expression level also decreased after miR-9-5p inhibition (Fig. 3h, i).

### *Leptin* is a target gene of miR-9-5p

**Fig. 4:**
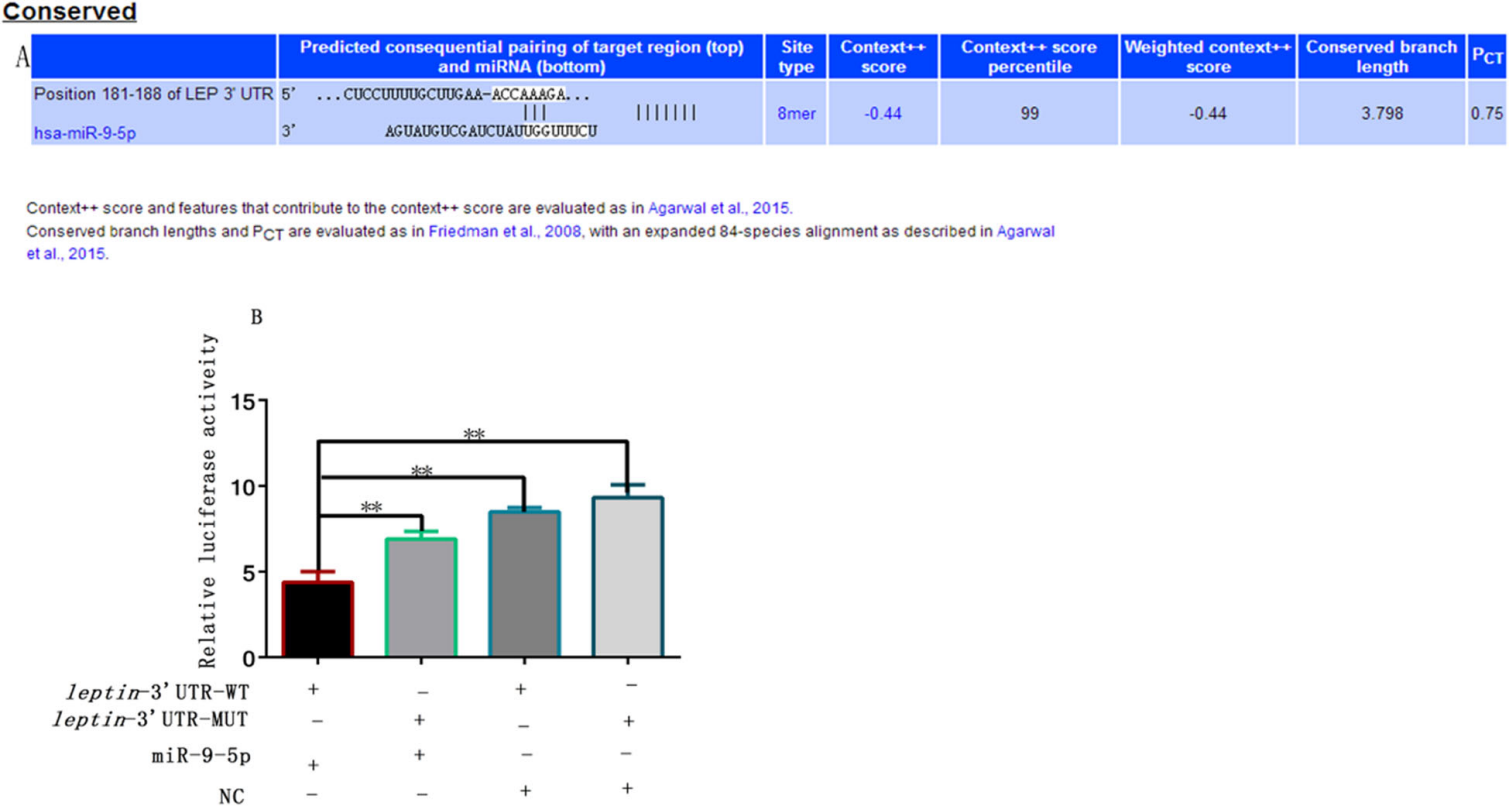
*Leptin* is a target miR-9-5p gene. **a** prediction of target gene (**b**) PmirGLO and pmirGLO-*leptin* 3′ UTR were cotransfected with the mimic into Hela cells, and normalized firefly luciferase activity was determined (*n* = 3). (** *P* < 0.01)

To further determine the function of miR-9-5p, bioinformatics analysis was carried out to predict direct targets of miR-9-5p. MiRWalk2.0 (http://zmf.umm.uni-heidelberg.de/apps/zmf/mirwalk2/) software revealed that *leptin* contains the target sites for miR-9-5P (Fig. 4a). To verify the existence of direct binding sites between miR-9-5p and *leptin*, the luciferase reporter assay was carried out and the results showed that the firefly luciferase activity significantly reduces during co-transfection of pmirGLO-LEPTIN-3′ UTR and miR-9-5p agomirs (Fig. 4b).

### MiR-9-5p downregulated *leptin* mRNA expression

**Fig. 5:**
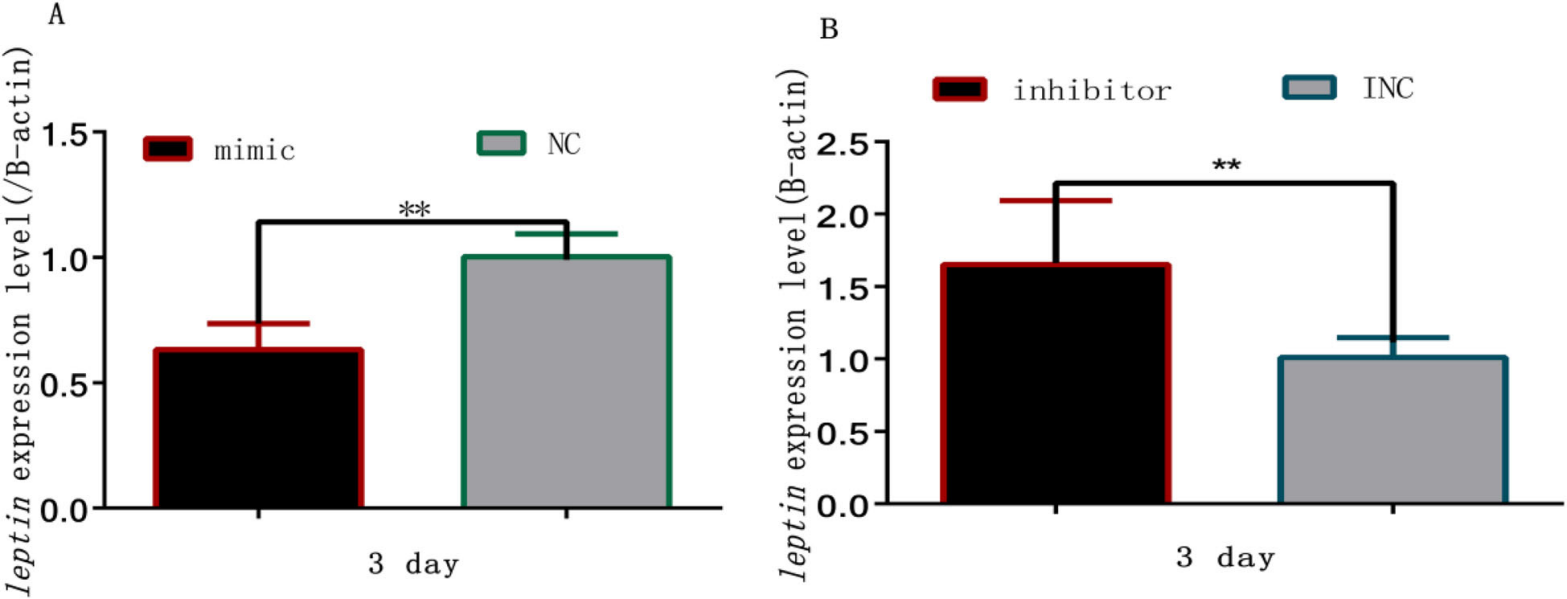
MiR-9-5p negatively regulated *leptin* expression: **a***Leptin* expression based on quantitative real-time polymerase chain reaction (QRT-PCR) during adipogenesis with miR-9-5p overexpression. **b***Leptin* expression based on QRT-PCR during adipogenesis with miR-9-5p knockdown. (“**”, *P* ≤ 0.01)

To better understand the role of miR-9-5p during preadipocyte differentiation, we further validated the effect of miR-9-5p on the expression of *leptin*. As shown in Fig. 5a, overexpression of miR-9-5p led to an obvious decrease in the mRNA levels of *leptin*. In contrast, when the endogenous miR-9-5p was inhibited with the miR-9-5p inhibitor, the expression of *leptin* increased as compared with the NC group (Fig. 5b).

### Downregulation of *leptin expression* promoted rabbit preadipocyte differentiation

**Fig. 6:**
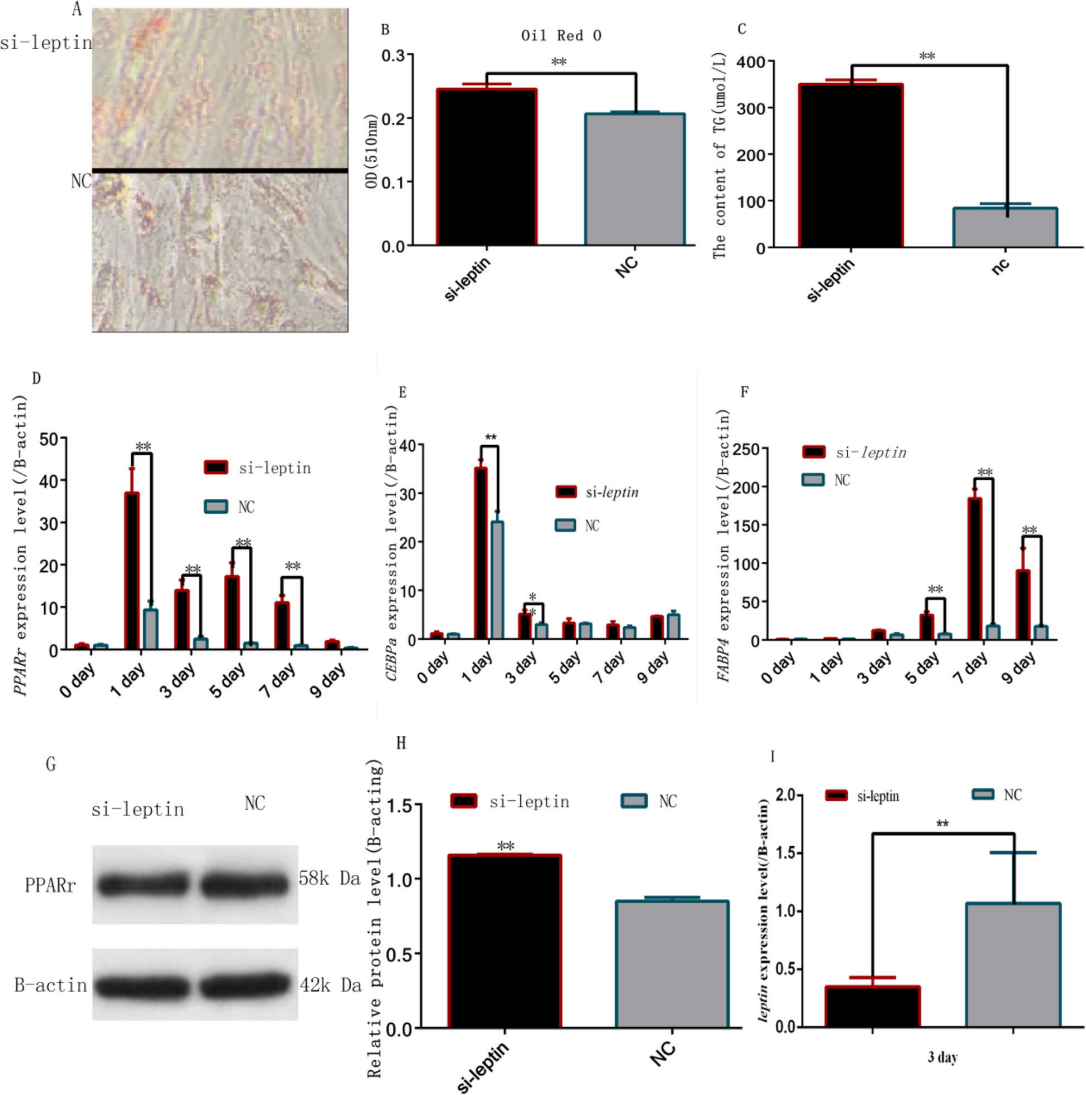
Inhibition of *leptin* gene promoted rabbit preadipocyte differentiation (**a**) Oil Red-O staining of lipid droplets on day 9; (**b**) Quantitative detection of oil red O staining on day 9; (**c**) Accumulation of triacylglycerol on day 9; (**d**) *PPARγ* expression levels during preadipocyte differentiation after transfecting with si-*leptin* and NC; (**e**) *CEBPα* expression levels during preadipocyte differentiation after transfecting with si-*leptin* and NC; (**f**) *FABP4* expression levels during preadipocyte differentiation after transfecting with si-*leptin* and NC; (**g**, **h**) *PPARγ* protein levels during preadipocyte differentiation after transfecting with si-*leptin* and NC. **i***Leptin*. expression levels during preadipocyte differentiation after transfecting with si-*leptin* and NC (“*”, *P* ≤ 0.05; “**”, *P* ≤ 0.01)

To further confirm whether *leptin* is involved in the differentiation of rabbit preadipocytes, si-*leptin* was utilized to knockdown endogenous *leptin*. We found that the accumulation lipids and triacylglycerol accelerated in the preadipocytes cells (Fig. 6a, b, c). When *leptin* was knocked down, the mRNA levels of *PPARγ* and *C/EBPα* rapidly increased on the first and third day after transfection, whereas *FABP4* rapidly increased on the 5th and 7th (Fig. 6d, e, f). Furthermore, *PPARγ* protein expression level significantly increased in si-*leptin* group than NC group, 3 days after transfection (*P* < 0.05) (Fig. 6g, h). However, after knocking down endogenous *leptin,* the expression levels declined rapidly (Fig. 6i).

## Discussion

In recent years, miRNAs have been reported to be expressed in mammalian adipocytes and play an important role in the regulation of adipogenesis [39]. Previous studies have discovered a functional interplay between glucose-dependent insulin secretion, mir-9 levels, and *Sirt1* protein in pancreatic islets β--cells [30]. It has also been found that miR-9 and *Sirt1* are involved in glucose-dependent insulin secretion in islet β cells, and it has been proved that *Sirt1* is a target gene of miR-9 [27]. In addition, miR-9-5p could negatively regulate *Sirt1* expression and plays an important role in the regulation of cell proliferation and migration [29]. *Sirt1* can bind to two coenzyme factors of *PPARγ*, which inhibit the binding of *PPARγ* to the target gene and the differentiation of adipocytes [25]. It has also been shown that miR-9-5p promotes the secretion of Granuphilin-a and reduces the secretion and synthesis of insulin by targeting *Onecut2* [26]. The results showed that PPARγ ligand enhanced glucose uptake in adipose tissue by enhancing the expression of GLUT-4, which can improve insulin sensitivity [13]. Furthermore, *PPARγ* is a marker gene of preadipocyte differentiation. So, miR-9-5p may regulate the differentiation of adipocytes.

The triglyceride content was found to be more and lipid droplets were larger after transfection of miR-9-5p. The upregulation of miR-9-5p increased the expression of adipogenic marker genes *FABP4*, *PPARγ,* and *CEBPα*, whereas, the downregulation of miR-9-5p using the synthetic inhibitor markedly reduced the formation of neutral lipid droplets and suppressed the expression of marker genes at both the *PPARγ* mRNA and protein levels. These results suggest that miR-9-5p can promote the differentiation of rabbit preadipocytes. Previous studies demonstrated that NADPH oxidase 4 (*NOX4)* is a target gene of miR-9-5p [8] and *NOX4* is the key enzyme of reactive oxygen species (ROS). It was reported that insulin release is inhibited by increasing ROS in islet β-cells [3, 5] and the content of *leptin* is positively correlated with insulin [2, 24]. In the present study, a dual luciferase reporter assay was employed to validate that *leptin* is the target gene of miR-9-5p. The results suggested that miR-9-5p can negatively regulate *leptin* gene expression. Previous studies have shown miR-9-5p can regulate secretion and synthesis of insulin. Insulin can stimulate the secretion of *leptin* by increasing the mRNA expression of *leptin*, and in turn, *leptin* can directly inhibit the secretion of insulin [7]. Studies have shown that insulin can improve the differentiation rate of preadipocytes and promote the accumulation of fat [33]. It has also been found insulin may affect the expression of *PPARγ* by affecting sterol regulatory element-binding protein-1c (*SREBP-1c*) [11]. *Leptin* can also regulate the differentiation of adipocytes and previous studies have demonstrated that *leptin* can inhibit the synthesis of lipids and promote the decomposition of triglycerides in adipocytes [9]. So, miR-9-5p may target *leptin* to promote the differentiation of rabbit preadipocytes.

*Leptin* is secreted by adipocytes and the mRNA level of *leptin* decreases at a later stage of adipocyte differentiation [1]. Previous studies have shown that *leptin* is involved in a local ultra short negative feedback loop that regulates the differentiation of preadipocytes [36]. Literature also suggests that *leptin* suppresses specific biochemical processes, which contribute to lipid accumulation and adipocyte differentiation [34, 40]. Furthermore, *leptin* can accelerate the proliferation of preadipocytes, inhibit the synthesis of lipids and promote the decomposition of triglycerides [9]. In the present study, we found that the suppressed expression of *leptin* dramatically promotes the differentiation of preadipocytes. This finding is in agreement with a previous studie which suggested that *leptin* inhibits the differentiation of preadipocytes. Therefore, we speculated that miR-9-5p positively regulates rabbit preadipocyte differentiation by inhibiting *leptin*. However, the biological basis and mechanism of this phenomenon need further research to get rabbits of high intramuscular fat content. In recent years, numerous studies have been motivated by the desire to get animals (e.g., cattle and pigs) with high intramuscular fat content, but have not yielded satisfactory outcomes. This is largely due to the fact that there is a need to increase the body fat and muscle fat in the same animal at the same time. However, rabbits are animals with particularly low-fat content. So, we can increase the body fat and intramuscular fat at the same time, and this makes it possible to grow rabbits with high intramuscular fat content.

## Conclusion

In summary, our data demonstrated that miR-9-5p can promote the differentiation of rabbit preadipocytes and *leptin* acts as one of the downstream targets of miR-9-5p. In addition, knockdown of *leptin* promoted adipogenic differentiation. Altogether, we conclude that miR-9-5p promotes rabbit preadipocyte differentiation by suppressing the expression of *leptin* mRNA.

## Data Availability

All data generated or analyzed during this study are included in this published article.

## Abbreviations

FABP4: Fatty acid-binding protein 4
C/EBPα: CCAAT-enhancer binding protein α
PPARγ: Peroxisome proliferator activated receptorγ
ASCs: Adipose tissue-derived stem cells
MAPK2K3: Mitogen-activated protein kinase 2/ kinase 3
3 T3-L1: Mouse Embryonic mouse fibroblast
PBS: Phosphate-buffered saline
NOX4: NADPH oxidase 4
ROS: Reactive oxygen species
SREBP-1c: Sterol regulatory element-binding protein-1c
